# Dizziness in older people: at risk of shared therapeutic nihilism between patient and physician. A qualitative study

**DOI:** 10.1186/s12875-016-0474-3

**Published:** 2016-07-16

**Authors:** Hanneke Stam, Marjanne Wisse, Bram Mulder, Johannes C. van der Wouden, Otto R. Maarsingh, Henriëtte E. van der Horst

**Affiliations:** Department of General Practice and Elderly Care Medicine and EMGO Institute for Health and Care Research, VU University Medical Center, 1081 BT Amsterdam, The Netherlands

**Keywords:** Dizziness, Aged, Impairment, Qualitative research, General practice

## Abstract

**Background:**

Dizziness-related impairment is a strong predictor for an unfavourable course of dizziness in older people. In this study we explored the experiences of older patients with significant dizziness-related impairment and their wishes and expectations regarding general practitioner (GP) care. Knowing the expectations and priorities of people with dizziness may enable the GP to provide tailor-made care, which in turn may substantially increase the quality of life and decrease the use and costs of health care.

**Methods:**

We conducted a qualitative study with semi-structured interviews. We selected patients from ten Dutch general practices. Patients were invited to participate in the study if they were ≥ 65 years, visited their GP because of dizziness and were significantly impaired due to dizziness (Dizziness Handicap Inventory ≥ 30). We applied content analysis to the semi-structured interviews.

**Results:**

Thirteen participants participated, seven were female. Analysis of the interviews resulted in the overall theme “Dizziness in older people: at risk of shared therapeutic nihilism by the patient and the GP”. Firstly, this can explained by the fact that participants frequently presented dizziness as a secondary complaint when they visited the GP for another complaint. Secondly, participants reported that the GP often could not help them with any treatment. Despite a poor therapeutic outcome, the vast majority of participants was satisfied how the GP handled their dizziness. Yet, understanding the cause of dizziness seems important for dizzy older patients.

**Conclusions:**

Despite significant dizziness-related impairment, older dizzy patients may not present dizziness as main reason for encounter. Presenting dizziness as a secondary complaint may give GPs the - wrong - impression that the dizziness-related impairment is only mild. GPs need to be aware of this potential underreporting. Knowing the cause of dizziness seems important for older patients. Yet, GPs regularly did not succeed in identifying the underlying cause of dizziness. Therefore, GPs should manage the expectations of older dizzy patients regarding diagnosis and successful treatment, by informing them about the uncertainty and unpredictability of dizziness. We also recommend GPs to focus on improving functional ability; this is the key to escape from therapeutic nihilism by the GP.

**Electronic supplementary material:**

The online version of this article (doi:10.1186/s12875-016-0474-3) contains supplementary material, which is available to authorized users.

## Background

Dizziness frequently occurs, especially in older people [1–6]. One out of ten older patients visit their general practitioner (GP) at least once a year because of dizziness [1, 6]. Dizziness can refer to several sensations including a giddy or rotational sensation, a loss of balance, a faint feeling, light-headedness, instability or unsteadiness, a tendency to fall, or a feeling of everything turning black [7]. It is well-known that dizziness strongly affects daily living of older people [8–11]. Dizziness has moderate or severe impact on daily living of more than 60 % of older people with dizziness in primary care [7]. Furthermore, dizziness in older people is associated with increased depressive symptoms, lower self-rated health and reduced social activities [8, 12]. Dizzy older people also have an increased fall risk [13], potentially leading to impaired functioning and high health care costs. There is a broad etiologic spectrum of peripheral, central (neurological), and general medical causes for dizziness. However, it is often difficult for GPs to establish the origin of dizziness in older people [1, 6]. The broad etiologic spectrum of dizziness combined with a high rate of unknown cause of dizziness leads to a wide variety of management strategies by GPs [6].

A qualitative study design provides depth and detail of the impact of dizziness at an individual level instead of a group level and can introduce new topics not initially considered. Until now, only two studies explored the impact of dizziness on older people’s daily life in a qualitative way [11, 14]. Awareness of the limitations that older dizzy patients are facing, might enable the GP to better understand the barriers and needs of these patients. In addition, it is desirable to understand the wishes of older dizzy patients concerning GP care. So far, only few studies have investigated older dizzy patients’ experiences and wishes regarding GP care. Olsson Möller et al. reported that older dizzy patients preferred more information about the cause and prognosis of dizziness and also would like to have more examinations [11]. In a study by Kruschinski et al. some older dizzy patients felt that diagnostic efforts to find causes should be increased [14]. Furthermore, patients did not seem well informed about possible causes of dizziness nor about the therapeutic options [14]. Insights in the wishes and needs of older patients concerning GP care for dizziness may lay a basis for improved care for patients with dizziness.

This is the first qualitative study that focusses on significantly impaired older dizzy patients. Dizziness-related impairment is a strong predictor for an unfavourable course of dizziness [15], potentially resulting in a greater burden on individuals, health care systems and society. Improving care for this group may substantially increase the quality of life and could decrease the use and costs of health care. Therefore, the aim of this qualitative study is to explore the experiences of living with dizziness in older patients with significant dizziness-related impairment and their wishes and expectations regarding GP care for dizziness.

## Methods

### Design and ethical approval

This qualitative study was conducted using semi-structured interviews. The Medical Ethics Review Committee of VU University Medical Center approved the study (approval number: NL49604.029.14).

### Participants

For this study we selected participants from the ‘Reduction Of Dizziness in older pEOple’ (RODEO) study, an ongoing Dutch randomised controlled trial on the effectiveness of a multifactorial intervention for dizziness in older people. Details of the study design have been published elsewhere [16]. Participants from the RODEO study were included from 50 general practices in the Netherlands. They were invited to participate in the study if they were 65 years or over and visited their GP because of dizziness in the preceding 3 months. We defined dizziness as recurrent dizziness for at least one month, including a giddy or rotational sensation, loss of balance, faint feeling, light-headedness, instability, and/or tendency to fall [7]. Patients were only eligible to participate in case of significant dizziness-related impairment (Dizziness Handicap Inventory (DHI) score ≥ 30 points [17]). The DHI is a self-report questionnaire that assesses the disability associated with dizziness. DHI-scores range from 0 to 100 and can be classified into mild (0–30 points), moderate (31–60 points) and severe (61–100 points) perceived disability [18]. Patients suffering from severe cognitive impairment, acute psychiatric problems or terminal disease were excluded. Furthermore, patients who did not master Dutch were excluded.

For this qualitative study we invited RODEO participants who were receiving usual care from their GP. Patients participating in the intervention arm of the RODEO study were not approached. Potential participants for this qualitative sub-study were contacted by phone. If they had interest in participating, they received written information concerning the study. Prior to the start of the interview, the interviewer asked for written informed consent. We purposely selected a diverse group of participants based on gender, age, GP, marital status and duration and intensity of symptoms.

### Data collection

The interviews took place at the participants’ homes in March and April 2015. A medical doctor (HS) trained in qualitative research conducted the first two interviews accompanied by one of two last-year medical students (MW and BM). The remainder of the interviews were conducted by MW and BM, one being the main interviewer and the other being the observant. The interviewers had no professional relationship with the participants. They presented themselves as medical trainees. This created a non-hierarchical relation with the participant and allowed the participants to speak freely. A topic list was used as guidance for the interviews (see Additional file 1). The topic list covered all the topics that we were interested in, based on the aim of or study and on prior research [11, 14, 19]. The topic list was revised after the analysis of the first three interviews. The revision included changing the opening question and reordering of some of the topics. The interviewers avoided closed questions and encouraged participants to talk freely about their experiences of living with dizziness and their wishes and expectations regarding associated GP care. The interview length depended on the time that was required to discuss all topics from the list.

All interviews were audio recorded and transcribed verbatim. To increase validity, a summary of the interview was sent to the participant by post and the interviewer later phoned the participant for feedback (member check).

### Analysis

We applied inductive content analysis to the verbatim semi-structured interviews [20]. This analysis process includes open coding, creating categories and abstraction. Open coding consists of writing down codes in the text while reading an interview. A code is a word that covers the content of every sentence or group of sentences with the same subject. After open coding, all codes are sorted and grouped around central themes, creating categories. Abstraction is the last step of inductive content analysis, leading to the formulation of a general description of the topic [20].

Two of the authors independently coded the manuscript of each interview (MW and BM). A list of all codes was made after coding the first three interviews, this code list was updated after the coding of every subsequent interview. This enabled us to continue interviewing until saturation was reached, referring to the point where no new codes were obtained from subsequent interviews. Four authors (HS, MW, BM and OM) sorted and grouped the codes around central themes, creating categories. The four authors compared and discussed the codes and categories until consensus was reached. The coding process was supported by Atlas.ti 7.2 software package [21].

## Results

### Participants

A total of fifteen patients were invited to participate in the study. Thirteen patients were willing to participate and only two invited patients declined to participate. At the tenth interview no new information emerged. Thereafter, we performed three additional interviews, to ensure saturation was reached. The mean interview time was 25 min (range 19–40 min).

Table 1 presents the characteristics of the participants. The mean age of the participants was 78 years (range 68–92), seven participants were female (54 %). The mean DHI-score was 41 points (range 30–56). A DHI-score of ≥ 30 points corresponds to moderate to severe dizziness-related impairment [17].

**Table 1:** General characteristics of participants

Participant	Gender	Age	Living situation	Self-rated health^a^	DHI score	Time since start	Dizziness frequency^b^	Dizziness duration^c^
1	F	92	alone	70	36	1–6 months	Daily	≥1 h
2	M	69	alone	52	30	2–10 year	Daily	1–10 min
3	M	85	alone	55	40	>10 year	Weekly	10 min - 1 h
4	M	75	with partner	70	36	2–10 year	Daily	≥1 h
5	F	70	with partner	80	32	1–6 months	Daily	1–10 min
6	F	78	alone	50	54	1–6 months	Daily	variable
7	M	82	with partner	70	36	2–10 year	Monthly	≥1 day
8	M	82	alone	40	56	2–10 year	Daily	10 s - 1 min
9	M	79	alone	60	52	>10 year	weekly	≥1 h
10	F	84	alone	100	48	>10 year	Weekly	≥1 h
11	F	76	with partner	50	38	1–6 months	constantly	variable
12	F	68	with partner	90	30	2–10 year	Monthly	10 min - 1 h
13	F	79	alone	60	42	1–6 months	Monthly	<10 s

Abbreviations: *DHI score*, dizziness handicap inventory score (0–100), *F* female, *M* male, *mo.* month, *yr* year, *hr* hour, *min* minute, *sec* second

^a^Self-rated health (scale 0–100) as measured with EQ-5D-5 L VAS (http://www.euroqol.org/about-eq-5d/how-to-use-eq-5d.html)

^b^One or more dizziness attacks daily/weekly/monthly

^c^Duration of one dizziness attack

### Themes

In this study we investigated the impact of dizziness in everyday life and the patients’ wishes and expectations of GP care for dizziness. Dizziness in older age is a symptom at risk of shared therapeutic nihilism by the patient and the GP resulting in de overall theme of this study: *Dizziness in older age: at risk of shared therapeutic nihilism by the patient and the GP*. This overall theme constitutes two themes with subsequent subthemes:

1. Downplaying dizziness
  *Restrictions and psychological consequences*
  *Dealing with dizziness without help*
2. Patient’s positive fulfilment of GP care
  *Patients’ nihilism regarding GP care*
  *Pleased with GP care despite GP nihilism*
  *Needs concerning GP care*

1. **Downplaying dizziness**

*Restrictions and psychological consequences*

As expected, all participants faced barriers in daily life as a result of dizziness. Almost every participant experienced restrictions in mobility. Many participants felt that dizziness negatively influenced their range of action because they had difficulties with walking, were not able to drive a car or bike, or had difficulties using the public transportation.

*Quote P13: “And, umm, yeah, you obviously shouldn’t take any risks. And that’s the reason I don’t cycle anymore. And also why I don’t drive anymore, so I won’t hurt anyone. That wouldn’t be sensible, putting other people at risk”.*

Mobility restrictions also influenced work in and around the house. The majority of the participants reported difficulties with housekeeping and other domestic tasks. Not being able to climb a ladder was often mentioned as cause for not being able to perform tasks in and around the house.

*P8: “Oh, I’m too scared to stand on a ladder. […] That umm, triggers it I guess. Having no horizon or something.”*

*I: “And what do you do if you have to use a ladder?”*

*P8: “I don’t use it”.*

Restrictions in hobbies were often mentioned by participants. Hobbies that were most often cited as being affected by dizziness, were going for a walk, practicing sports and gardening. Furthermore, dizziness often reduced their participation in social activities. For example, participants did not visit family meetings anymore, because they could not handle a crowd. In addition, participants did not go on holidays and experienced difficulties with other social activities such as volunteering.

*P5: “I had to cancel my birthday party. As it was my birthday, so I said, I can already see myself moving around with my coffee and my cake. I said ‘Well guys, forget it. Go ahead, just cancel the whole thing”.*

Many participants described a continuous feeling of insecurity in everything they did. They felt insecure while suffering from dizziness, but they also felt insecure after the dizziness vanished because they felt that it could potentially recur at any moment. Some participants reported that they were anxious to fall.

*P3: “…because of the insecurity, with the dizziness and the unsteadiness etc. […], I’m too scared to go somewhere the last couple of years and so I don’t go anywhere”.*

Although they reported many restrictions in daily life, most participants stated that living with dizziness was not a major issue. Some participants reported they were struggling with their dizziness; others described they were afraid of having a serious disease and to pass away and others were feeling down and did not have any hope that the dizziness would ever go away.

*P10: “I have to do everything at a slower pace. Well, that’s not that bad, at my age, to have to do things at a slower pace”.*

*P5: “So I got the impression that there is something seriously wrong with me. What’s happening in my head, why does this keep happening?”*

*Dealing with dizziness without help*

All participants reported strategies to anticipate possible consequences of dizziness, such as falling. The majority of the participants described they were carefully walking through their houses. Many participants reported that prior to setting themselves into motion, they had to focus and consciously plan their steps. Furthermore, various participants described how they deliberately leaned on furniture while moving through their house. Almost half of the participants mentioned they were using aids to cope with dizziness. For example by using a wheeled walker (rollator), walking aid or by traveling by taxi instead of driving a car.

*P9: “I do have to think about it really hard every day. I can’t do anything out of the ordinary. Can’t bend over all of a sudden. Of course you know that, well, you find out yourself. Because then you fall, if you do those kind of things. So I don’t do that”.*

Some participants clung on to their hope that the dizziness would go away at some point. Also, many participants abided by having dizziness and were trying to accept it. Participants mentioned they were trying to focus on activities they could still do instead of focussing on limitations due to dizziness. Moreover, almost half of the participants reported they got used to experiencing dizziness. A lot of participants also said that dizziness complaints were not as disabling as other chronic diseases or health issues among acquaintances.

*P6: “Well. How do you deal with it? Just accept it. That’s it. You can’t deal with it. You have to accept it. There are so many things that aren’t fun anymore”.*

*P13: “I can deal with it in this way. I keep thinking, some people have a bad hip, other people have a bad knee. And other people, whatever. My friends, they all have something. It hurts if I press here, it hurts there. Well, then I think, I have something as well. And yes, that’s how I accept it”.*

2. **Patient’s positive fulfilment of GP care**

*Patients’ nihilism regarding GP care*

Most participants consulted the GP because of dizziness when they faced difficulties with getting out of bed or with walking; hence the severity of the symptoms was a reason to consult the GP. Yet, they rarely presented dizziness as the main reason to visit their GP. Dizziness was mostly presented as a secondary complaint, while visiting the GP for another complaint.

*P12: “And when do you do something about it, when you’re at the doctor for another reason. And then you think oh yeah, now that I’m here anyway, I bring up my dizziness”.*

Participants considered their dizziness as not sufficiently serious as a main reason for encounter with their GP or they expected that the GP would not be able to help to relieve their symptoms.

*I: “Is there a reason why you never consulted the GP for your dizziness?”*

*P13: “Well, I’m uhm, I’m getting older. You can’t do everything like you did it 10 years ago. You just have to deal with it.”*

*Pleased with GP care despite GP nihilism*

Many participants were very pleased about the way the GP handled their dizziness. They felt the GP took their complaint seriously and had sufficient time for them.

*P6: “So when I came to him, then I said: ‘Doctor, this will be a puzzle, won’t it? ‘Yes’, he says, ‘Indeed’. And then he will get to the bottom of how he can help me. No, this GP is dealing with it very well. I can’t say anything else.”*

Only one participant felt he was not taken seriously by the GP for his dizziness.

*P2: “And if you start talking about these things, then sometimes you just see those eyes drift away into the distance, and you see her thinking what shall I have for dinner tonight. […]. They don’t really care. Haha. Yeah, that’s just the way it goes. But that doesn’t mean that I haven’t been taken seriously by her in all those 10 years”.*

A lot of participants reported that the GP initiated a treatment, e.g., prescribed medication or referred the participant to a physiotherapist or medical specialist. Participants were satisfied with these management strategies. However, once they found out the therapy did not relieve their symptoms of dizziness, participants reported that the GP often could not help them with any other treatment. The GP sometimes explained that dizziness is part of the ageing process with nothing much to do about it.

*P5: “The GP told me it is was an inflammation of the vestibular system or something. I asked him ‘So what do we do now?’, and he responded, ‘Nothing’.*

*P7: “Well the GP visited me at home and told me what to do. And for the rest, well uhm, nothing really happened.”*

Some participants were disappointed the GP was not able to help them and felt that GP care for dizziness was not useful. Few participants doubted if the GP had thorough knowledge of dizziness.

*P11: “I mean, he can’t do anything, he doesn’t know anything about dizziness”.*

Nonetheless, there were also participants who had sympathy for the efforts of the GP and accepted that the GP could not cure their dizziness.

*P12: “I can’t blame the GP, for instance that he should have done more to help me or whatever. No, I have no clue how else he could have been able to help me.”*

*Needs concerning GP care*

All participants were asked what the GP could have done better in handling their dizziness. The vast majority of the participants were satisfied with the way the GP handled their dizziness. Some participants would give the GP the recommendation to improve the care for dizziness. Frequently, the advice to do more additional testing to find out the underlying cause of dizziness was reported. One participant would like to have received more information about which activities she was allowed to do. Also, a participant wondered if there were any risks involved with the dizziness and if there were special things to pay attention to.

*P2: “Yes, any kind of medical examination is pleasant. It gives hope for improvement”.*

## Discussion

To the best of our knowledge, this is the first qualitative study that focusses on significantly impaired older dizzy patients. Our study revealed that shared therapeutic nihilism by the patient and the GP may be a risk in dizziness in older age. As expected, participants described many dizziness related restrictions in daily life. However, participants seemed not to act accordingly: only in case of severe dizziness participants presented dizziness as main reason for encounter with their GP. Most participants presented dizziness as a secondary complaint when they visited the GP for another complaint. They felt that their dizziness was not serious enough to present as main reason for encounter with their GP or they expected the GP would not be able to relieve their symptoms. After visiting the GP, the vast majority of the participants reported they were very satisfied with the way the GP handled their dizziness. Even though, according to the participants, the GP was often not able to reduce the dizziness symptoms or did not start any therapy at all.

The reported restrictions in previous qualitative research of both younger and older patients [19, 22], are in line with the reported dizziness related restrictions in this study. All participants reported strategies to anticipate on possible consequences of dizziness, such as falling. Examples of these strategies are carefully moving through the house or concentrating and determining where to go prior to moving. These self-learned strategies are also described in other qualitative studies on patients with chronic dizziness [11, 14, 19].

Some participants reported they cling on to the hope the dizziness will disappear someday. Yet, the majority of participants seem to be able to cope with dizziness; they have got used to having dizziness, they focus on activities they still can do or they feel dizziness is not as disabling as their other chronic diseases or health issues among acquaintances. Previous studies also report dizzy patients who accept the presence of dizziness [14], have feelings of gratitude over things they are still able to manage [11] and assert their ability to cope with the dizziness and to carry on as normal [23].

It is remarkable that participants of this study stated their dizziness was not severe, even though they reported many restrictions in daily life. This seems contradictory. Yardley and Beech explained this apparent inconsistency in terms of the social roles that those with chronic illness have to fulfil: they must provide evidence of their illness to elicit sympathy and assistance but they must also affirm their personal competence to sustain social dignity and independence [23]. In a study on coping with multimorbidity in older age, Löffler et al. described a “*can-do-approach to life*”: patients activated all their resources for the continuation of a meaningful and autonomous life [24]. In older dizzy aged patients, seeking help form their GP may not fit into their fight for an autonomous life.

Therapeutic nihilism by the older patient was revealed by the fact that dizziness was rarely the main reason for visiting the GP. This was unexpected given the high impact of dizziness in everyday life experienced by these participants (as reported during interviews and according to their DHI score of ≥30 points, corresponding to significant dizziness-related impairment [17]). Instead, participants mostly presented dizziness as a secondary complaint when they visited the GP for another complaint. Presenting dizziness as a secondary complaint may give GPs the - wrong - impression that the dizziness-related impairment is only mild. Other studies in older patients with chronic dizziness did not report that patients rarely visit their GP for dizziness [11, 14]. Many participants in this study described that they felt the dizziness was not serious enough to consult their GP for. The fact that a lot of participants described that the dizziness complaints were not as disabling as other chronic diseases or health issues among acquaintances, might also have attributed to not seeking help. This fits into non-help-seeking behaviour of older people with other health complaints. Previous studies revealed that older patients did not seek help because they attributed their symptoms to normal aging, they had the notion that their symptoms were to mild or not serious enough, or they prioritized help-seeking for more serious comorbid conditions [25–27].

Participants were very satisfied with the care they received for their dizziness, despite the therapeutic nihilism by the GP. However, some of these satisfied participants also reported they were disappointed that the GP could not relieve their dizziness symptoms. Others felt that their GP did not have a thorough knowledge of dizziness. And finally, some preferred more additional testing to find out the underlying cause of dizziness. In general, older people are very satisfied with their GP [28]. Furthermore, Chang et al. found that ratings of healthcare were not associated with technical quality of care but instead were strongly associated with ratings of communication in a sample of frail older patients [29]. This might explain why older dizzy participants in this study reported they were very satisfied with the care for dizziness despite their discontent with some specific aspects of this care. In line with Olsson Möller et al., participants in this study experienced that treatments sometimes had no or only temporary effects [11]. Nevertheless, a priority of older dizzy patients is to at least stabilize the symptoms and preserve mobility through prevention of falls [14].

Participants reported they would like more additional testing to find the cause of dizziness. This desire to know the origin of dizziness has also been reported previously [11, 14, 19]. In a study with younger dizzy patients, the inability of the physician to find any proof of disease was experienced as very frustrating and degrading [19]. For a significant proportion of older patients, doctors are unable to find an underlying cause of dizziness [1, 6, 30]. An unknown cause of dizziness can result in therapeutic nihilism by the GP. To increase the quality of life, we recommend to focus on improving functional ability when the cause of dizziness is uncertain and/or when subsequent treatment does not decrease dizziness symptoms. Focussing on improving functional ability is the key to escape from therapeutic nihilism by the GP. Furthermore, Drachman has recommended several treatment strategies for dizziness, including patient education to address the uncertainty and unpredictability associated with chronic dizziness [31]. We endorse this recommendation.

As all qualitative research this study is explorative. The data originate from a small sample of the total population with dizziness. Nevertheless, we endeavoured to maximise the trustworthiness of this study: we purposely selected participants with diverse characteristics; the two interviewers were non-medical which allowed the participants to speak freely; a topic list was used to guide the interviews; interviews continued until saturation was reached; data was analysed independently by two authors; categories were created in discussion sessions with four authors; all participants were asked for a member check.

## Conclusions

This study revealed that shared therapeutic nihilism by the patient and the GP is a risk in dizziness in older age. Significantly impaired older dizzy patients experienced a lot of restrictions in everyday life. Nevertheless, participants regularly felt their dizziness was not serious enough to present as the main reason for encounter with their GP. If they mentioned dizziness to their GP, participants mostly presented it as a secondary complaint when visiting the GP for another complaint. This may give GPs the - wrong - impression that the dizziness-related impairment is only mild. GPs need to be aware of this and should actively ask the dizzy patient about experienced restrictions to enable assessing of dizziness-related impact on everyday life. Furthermore, finding the cause of dizziness seems important for patients suffering from dizziness. Yet, GPs regularly did not succeed in identifying the underlying cause of dizziness. The GP should manage the expectations of dizzy patients regarding diagnosis and successful treatment, by informing them about the uncertainty and unpredictability of dizziness. To increase the quality of life, we recommend to focus on improving functional ability when the cause of dizziness is uncertain and/or when subsequent treatment does not decrease dizziness symptoms. Focussing on improving functional ability is the key to escape from therapeutic nihilism by the GP.

## Abbreviations

DHI, dizziness handicap inventory; GP, general practitioner

## Additional file

Additional file 1: Topic list (interview guide). (DOC 27 kb)
