# Supplementary material for: Dizziness in older people: at risk of shared therapeutic nihilism between patient and physician. A qualitative study
Source: BMC Fam Pract. 2016 Jul 16;17:74. doi: 10.1186/s12875-016-0474-3 (PMC4947269; doi:10.1186/s12875-016-0474-3)
Supplement: Additional file 1: — Topic list (interview guide). (DOC 27 kb) [file 12875_2016_474_MOESM1_ESM.doc]

**Everyday life**

*Opening question* When was the last time you experienced dizziness?

Additional questions What were you doing at that time? How did you feel? How did you handle this attack of dizziness?

*Barriers* Are there things you are not able do anymore as a result of suffering from dizziness?

*Facilitators* Do you have any strategies to reduce your dizziness symptoms?

*Help* Do you need help in daily life from others because of your dizziness?

*Social* How did family and friends react on your problems?

**GP care**

*Reason for encounter* Can you tell me when you consulted your GP because of dizziness? What was the reason you consulted your GP back then?

*Perception of GP care* How did you experience the care of the GP regarding dizziness?

Did the GP find an explanation for your complaints? How do you feel about this explanation?

*Improvement GP care* Are you satisfied with the way the GP handled your dizziness?

What went well? What could have been better?
